# Analysis of Phenolic Compounds for the Determination of Grafts (in) Compatibility Using *In Vitro* Callus Cultures of Sato-Zakura Cherries

**DOI:** 10.3390/plants10122822

**Published:** 2021-12-20

**Authors:** Dragana Skočajić, Uroš Gašić, Dragana Dabić Zagorac, Marija Nešić, Živoslav Tešić, Mekjell Meland, Milica Fotirić Akšić

**Affiliations:** 1 Department of Landscape Architecture and Horticulture, Faculty of Forestry, University of Belgrade, 11030 Belgrade, Serbia; dragana.skocajic@sfb.bg.ac.rs (D.S.); marija.nesic@sfb.bg.ac.rs (M.N.); 2 Department of Plant Physiology, Institute for Biological Research “Siniša Stanković”, University of Belgrade, 11060 Belgrade, Serbia; uros.gasic@ibiss.bg.ac.rs; 3 Innovation Centre of Faculty of Chemistry Ltd., 11000 Belgrade, Serbia; ddabic@chem.bg.ac.rs; 4 Faculty of Chemistry, University of Belgrade, 11000 Belgrade, Serbia; ztesic@chem.bg.ac.rs; 5 NIBIO Ullensvang, Norwegian Institute of Bioeconomy Research, Ullensvangvegen 1005, N-5781 Lofthus, Norway; 6 Department Fruit Science and Viticulture, Faculty of Agriculture, University of Belgrade, 11080 Belgrade, Serbia; fotiric@agrif.bg.ac.rs

**Keywords:** *Prunus*, callus fusion, staining, histology, polyphenols

## Abstract

The aim of this study was to prove that under *in vitro* conditions, the adhesiveness of the callus between rootstock and scion, the development of callus cells at the points of fusion, and the presence of phenolic components are closely related to the level of (in) compatibility of the grafting combinations between *Sato-zakura* cherry cultivars (‘Amanogawa’, ‘Kanzan’, and ‘Kiku-shidare-zakura’) and commercial rootstocks. *Prunus avium*, *Prunus* ‘Colt’, *Prunus mahaleb* and *Prunus serrulata* were used as compatible and *Prunus serotina* and *Pyrus communis* ‘Pyrodwarf’ were used as two potentially incompatible rootstocks. The results indicated the significant manifestations of the early signs of the incompatibility on the callus junction. Phenols, as well as tissue senescence, were very precisely localized by toluidine blue and alcian blue as well as safranin staining, which can indicate the early signs of the callus incompatibility in some grafting unions. In the callus unions of *Prunus avium* with ‘Amanogawa’ and ‘Kiku-shidare-zakura’ the results of chemical analyses indicated that the existence of several flavonols, flavones and phenol acids could be involved in the incompatibility process in grafted combination. The detection of flavonol astragalin in the unions can be a biomarker of compatibility between scion and the rootstock, while some polyphenols, such as neochlorogenic acid, sinapic acid, ellagic acid, caffeic acid, baicalein, naringenin, apigenin and luteolin can be used as the indicators of graft incompatibility. *p*-coumaric acid and ferulic acid could be used for detection of delayed incompatibility.

## 1. Introduction

Compatibility between grafting components can be described as creating strong connection among rootstock and scion and making vital and functional composite plants [1]. Formation of graft unions rely on numerous biochemical and morphological processes which encompasses callus formation and creating of a new active vascular tissue and cambium that will be formed across the graft [2]. Very often the grafting is done between representatives of the same species, genus, or both, but sometimes distant grafting partners can be used too. When connecting genetically divergent plants, incompatibility can be observed due to the different morphology, anatomy, physiology and ploidy level of the components, which all together influence the translocation of nutrients and water conductance [3,4]. On the other hand, such kinds of unions can be beneficial for the scion, making it more resistant to many biotic and abiotic stresses [5].

Research in the field of graft compatibility is mostly related to species that are of great importance for the field of utilitarian horticulture in vegetable crops [6,7,8], fruit production [9,10,11,12] and viticulture [13,14,15,16]. There are significantly more papers dealing with physiological, histochemical, anatomical and other aspects of tissue matching of grafting components in ornamental woody species [17,18]. A special group of trees, which is at the transition between utilitarian and ornamental horticulture, consists of decorative taxa of Japanese flowering cherries from the *Sato-zakura* group, which have a special significance for landscape architecture and horticulture [19]. As most of them were obtained through heterovegetative propagation, a potential field of research is the compatibility of the most common cultivars of ornamental cherries with the rootstock in a temperate climate. Hartmann et al. [20] noted that in addition to the common cherry (*P. avium*), vegetative selections of *Prunus avium* F12-1 and generative rootstock of the Japanese cherry *Prunus serrulata* can be used. Macdonald [21] recommended ‘Colt’ particularly because of its property of being resistant to *Thielaviopsis basicola* and *Agrobacterium tumefaciens*. Świerczyński and Stachowiak [22] successfully grafted cultivar ‘Amanogawa’, ‘Kanzan’ and ‘Kiku-shidare-zakura’ with ‘Colt’ as a rootstock. However, Duta [23] indicated a frequent occurrence of shoots from the rootstock that quickly outgrow the scion and that incompatibility in grafting ornamental cherries can be a significant problem. 

The *in vitro* method makes it possible to determine the potential incompatibility of a rootstock and a scion, in a relatively fast way. By analyzing the processes that take place on the callus joint, it is possible to assess the strength of the connection between the two components of grafting [24,25,26]. The arrangement of parenchymal cells, staining of cell walls as well as the accumulation of phenolic compounds can be determined by histochemical and anatomical analyses of the cells of the junction. At the same time, obtained *in vitro* results are correlated with those from in vivo, from the field conditions [26]. The study of cellular processes in the graft union goes in the direction of determination and quantification of metabolic profiles (products of primary and secondary metabolites) in cells and tissues [27,28]. Concentration of polyphenolic compounds and interactions of combined metabolites in the parenchymal cells of the rootstock and scion is closely related to specific genetic interactions between cells in grafted partners [29]. 

The great importance of such research for nursery production and the possibility of selecting the best combinations for grafting, were the reason for this research. Compounds that have an undoubtedly important role in grafting are phenolic compounds. The role of the secondary metabolism products, primarily polyphenolic compounds, is related to different stages of the grafting process in response to physical tissue injury [15] and the risk of pathogens penetrating the incision site and the reaction at the junction of two different tissues [30,31]. Although phenolic compounds do not play a role in the primary metabolism of plant growth and development [32], they participate in the communication of the plant with the environment and their quantity depends on environmental conditions. In response to drought, soil salinity, pathogen attacks or tissue injuries as various forms of stress, the production of phenolic compounds in plant tissues increases [33]. According to the mentioned role that polyphenolic compounds play in grafting, early determination of grafting incompatibility in rootstock and scion tissues by means of polyphenolic compound analysis [34,35,36] proved to be, by utilizing histological studies [9,37], isozyme analyses [38,39] and *in vitro* techniques [24,40], a relevant and suitable method for determining the functionality of the junction of two plant tissues.

The primary aim of this paper is to determine the polyphenolic compounds as a biomarker of the occurrence of incompatibility in callus union of the three *Sato-zakura* cherry and domestic cherry rootstocks and to explore whether such analysis can be a useful method for forecasting incompatibility in ornamental cherry trees. Further, this paper aims to compare histological and chemical callus tissues analyses of two callus partners as a good system to study the early response to cellular abnormalities associated with graft tissues when unions are formed. It is hoped that this research will contribute to a deeper understanding of whether callus fusion *in vitro* could be a useful and convenient method for early diagnosis of the graft incompatibility process in woody perennials, such as fruit crops or grapevines and herbaceous vegetable crops (watermelons, cucumbers, tomatoes).

## 2. Results

### 2.1. The Analysis of Anatomical Cross-Sections

**Figure 1:**
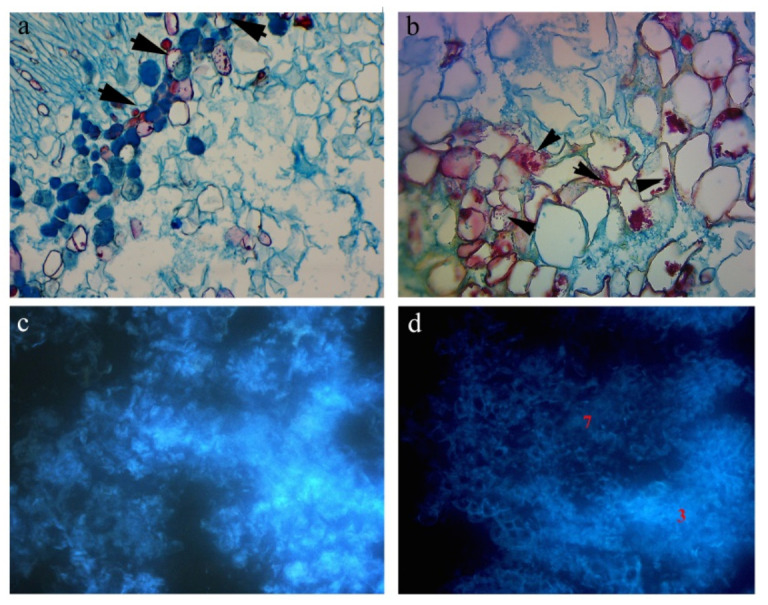
(**a**,**b**) Callus union interfaces between *P. mahaleb* and ‘Amanogawa’ (3.7) showing accumulation of phenol deposits at the union and enlarged cells. Thickened cell walls (black arrows) of *P. mahaleb* callus in incompatible grafts. (**c**,**d**) The response to the stain indicates the presence of phenolic compounds (arrows) in vacuoles in the callus cells ‘Colt’/‘Kanzan’ and *P. mahaleb*/‘Amanogawa’.

Incompatible graft unions, stained with toluidine blue, showed cell wall deflective and cells of irregular shape in the place of fusion (Figure 1a,b). Black arrows in section ‘a’ show cell wall thickening and the presence of phenolic compounds in vacuoles, while black arrows in section ‘b’ show the localization of phenols in callus samples (more intense staining of cells at the fusion part) which indicate incompatibility. The presence of phenolic compounds and their auto fluorescence ability as a response to ray absorption and the same time, the reemission of light of higher wavelength in incompatible fusions give the impression of brighter lighting versus dark field of view (Figure 1c,d). According to the review of studied fusions, the incompatibility occurs in 2.8, 3.7, 3.9, 5.8, 6.7, 6.8 and 6.9 unions. 

**Figure 2:**
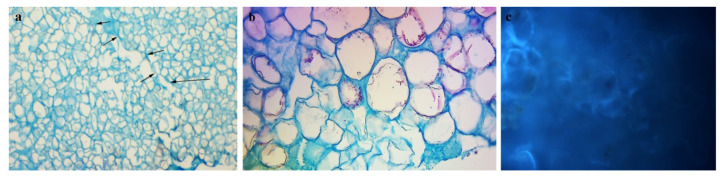
(**a**) Callus cells at the contact surface (arrows) of a compatible combination 21 days after callus fusion (**b**) the cells exhibit a homogeneous disposition; (**c**) low fluorescence in the callus cell union.

In all unions with *P. serrulata* rootstock (4.7, 4.8, 4.9), phenols were not synthesized on the fusions section or on the peripheral sides of the callus (exposed outside the agar) or fusions with the callus of another species (Figure 2a,b). In addition, according to the condition of the anatomical sections of the unions of Japanese cherries, combinations 2.7, 1.8 and 2.9 can also be considered compatible. *Prunus serrulata* ‘Kiku-shidare-zakura’ grafted on ‘Colt’ (2.9) could be classified as a high degree compatible combination. The rootstock of common cherry showed a high degree of compatibility only with the cultivar ‘Kanzan’ (1.8). For the other two (1.7 and 1.9), this combination proved to be partially successful and the histological analyses confirmed the existence of certain anomalies with the union.

The occurrence of partial incompatibility on temporary or permanent samples was determined for fusions 1.7, 1.9, 3.8, 5.7 and 5.9. By staining phenolic compounds, a lower share of these compounds (low fluorescence) was found in the callus of both rootstock and scion, mostly on the peripheral parts of the callus (Figure 2c). At callus cell unions, except for some cross-sectional sites, there were no signs of accumulation of phenolic compounds. Both methods for the identification of phenols (autofluorescence and toluidine blue staining) did not confirm the signs of the incompatibility of these taxa. 

### 2.2. Determination of Total Phenolic Content and DPPH Scavenging Activity

In Table 1, total phenolic component (TPC) and radical scavenging activity (RSA) comparison between homeoplastic and heteroplastic unions is presented. 

The mean values of TPC show a high degree of statistically significant differences in the content of total phenols of the graft unions. The content of total phenols varied from the lowest values recorded for the homeoplastic *P. serotina* fusion (0.19 g GAE/kg) to the highest one in *P. mahaleb* unions (from 2.27 (union mahaleb/‘Kanzan’) to 4.24 g GAE/kg (mahaleb/‘Amanogawa’)) and *Sato-zakura* junction with *P*. ‘Pyrodwarf’ (6.7 (2.14 g GAE/kg) and 6.8 (1.46 g GAE/kg)). Union 2.8 shows a single overlap and lower significant differences from unions 6.8 and 2.2. Unions 6.9, (1.34 g GAE/kg), 6.6 (1.29 g GAE/kg), 2.7 (1.205 g GAE/kg) and 2.9 (1.165 g GAE/kg) belong to the group of unions in which a higher content of TPC is induced. The lowest values of TPC with statistical significance were measured in homeo unions 5.5 and 1.1. Medium TPC values with a single overlap between groups (0.685–0.555 g GAE/kg) occur in all homeoplastic unions of *Sato-zakura* cultivars and their combinations with *P. serrulata*. The most uniform ratio of total phenol content was observed for the fusions of Japanese cherry cultivars (4.7, 4.8, 4.9). In the fusions with the *P. avium* and *P. serotina*, a significant increase in the content of total phenols was recorded in all fusions compared to homeoplastic ones. 

Radical-scavenging activity (RSA) was analyzed from the extract obtained from homeoplastic and heteroplastic callus fusions and their ability to remove DPPH radicals was tested. The results of RSA values ranged from 3.33–3.45 mmol TE/kg for all Japanese cherry to 16.25 mmol TE/kg in ‘Pyrodwarf’ heteroplastic callus unions. ANOVA analysis showed a statistically significant difference (*p* < 0.05) in the direction of increasing RSA in the junction of all combinations with *P. mahaleb* from 12.46 to 13.58 on unions 3.7, 3.8 and 3.9, and in ‘Pyrodwarf’ unions from 15.64 to 16.25 mmol TE/kg. Values of unions with ‘Colt’ (except 2.7) indicate significant higher values in comparison to *Sato-zakura* combination with *P. serotina* and *P. avium*. RSA values obtained for homeoplastic combination 9.9, 4.4 and 7.7, as well as combinations *P. serrulata*/‘Amanogava’, *P. serrulata*/‘Kanzan’ and *P. serrulata*/‘Kiku-shidare-zakura’, showed the lowest antioxidant activity. 

### 2.3. Analysis of Polyphenolic Compounds

The results of single phenol content in the callus tissue of *Sato-zakura* cherries united on different rootstocks calluses are reported in Table 2. A total of 19 compounds from 6 different phenolic classes were identified in the callus extracts. Among the detected flavonols, aesculin, rutin and hyperoside were detected in different concentrations in all unions. The highest levels of hyperoside were measured in the homeoplastic unions 6.6, 1.1 and 3.3. Furthermore, a significant increase in the concentration of hyperoside at the heteroplastic unions was identified at the unions 2.8 (‘Colt’/‘Kanzan’) and the unions 6.8 (‘Pyrodwarf’/Kanzan) with the values of 0.168 mg/L and 0.149 mg/L, respectively. The marked difference is not significant at 0.05 levels among *P. serrulata* cultivars 4.4, 4.7 and 4.8, and *P. serotina* combinations 5.5, 5.8 and 5.9. Among the remaining phenolic compounds in this group, an increase in concentration was observed for quercetin in the 2.9 and 2.8 unions with the ‘Colt’ rootstock (0.196 and 0.162 mg/L, respectively), ‘Pyrodwarf’ combinations 6.7 and 6.9 and *P. mahaleb* unions (3.7, 3.8, 3.9). No significant differences in quercetin amount were found between the mean values in *Sato-zakura* cherry combinations with *P. serrulata* (from 0.03 to 0.052 mg/L) and union 1.9. 

In the homeoplastic junction 3.3, the highest concentration of catechins was recorded (2.914 mg/L) but there was a decrease in concentrations of this polyphenolic compound in the heteroplastic combination union. However, there were no significant differences between recorded values in 3.9 and 3.3 unions. Furthermore, a significantly higher level of catechin was measured in combinations 1.8 and 1.7 (2.345 and 1.445 mg/L, respectively) than in homeoplastic unions 8.8, 7.7 or 1.1 (1.803, 1.27 and 1.009 mg/L, respectively).

Among the detected flavones, in some unions, cynaroside accounts for about 60% of the total content of all polyphenols quantified. In *P. mahaleb*, it was detected in the highest concentrations—in the homeo union (3.3)—reaching the value of 9.743 mg/L. However, in unions with all Japanese cherries the concentration of this compound decreased. A significantly higher concentration of cynaroside was measured in unions 1.9, 2.9 and 2.8 compared to homeoplastic union 1.1, 2.2, 8.8 and 9.9. For other flavones, the differences in the concentration were small—apigetrin occurs in constant concentrations from 0.012 to 0.013 mg/L in all unions and apigenin occurs in small concentrations with up to a 1% share in the total quantified polyphenols. Luteolin is detected in the homeoplastic union of ‘Kiku-shidare-zakura’ (9.9) with a value significantly lower than all heteroplastic union combinations (2.9, 3.9, 5.9, 6.9) except 4.9. For the other two cherry scions (7.7 and 8.8), concentrations of luteolin (0.162 and 0.205, respectively) in callus union with different rootstocks were higher only in unions 3.7 (0.187 mg/L) and 2.8 (0.212 mg/L).

According to the obtained measurements, a completely identical matrix of the ratio of increase or decrease of baicalein and naringenin in the unions of Japanese cherries and domestic rootstocks was recorded. The increase occurs in all three cultivars in unions with cultivars ‘Colt’ and *P. mahaleb*. 

*p*-coumaric acid is present in the lowest concentrations in all taxa of Japanese cherries, homeoplastic union 6.6 and heteroplastic unions 6.7 and 2.8. The highest increase in the content of this compound is recorded in the union 1.7 (0.216 mg/L), which was significantly lower than in homeoplastic unions 7.7 (0.146 mg/L) and 1.1 (0.063 mg/L). The unions with two-fold higher increase in regard to homeoplastic components were ‘Kiku-shidare-zakura’ unions 2.9, 5.9 and 6.9. 

As far as for other hydroxycynnamic acids, a significant increase in sinapic acid was obtained in the unions of all three cultivars of Japanese cherries with the cultivar ‘Colt’ (2.7, 2.8, 2.9). A similar phenomenon was observed for ‘Pyrodwarf’ combinations (6.7, 6.8, 6.9). The concentration of caffeic acid was not identified in Japanese cultivars and ‘Colt’ combinations. A significantly higher concentration of this acid was marked only in union 1.7. Ferulic acid indicated the similar pattern of distribution as for caffeic acid, but with higher mean values.

### 2.4. Principal Component Analysis (PCA)

The multivariate analysis is a dimensional reduction technique, which investigates, classifies and discriminates different samples based on their shared traits. In most cases it is used to determine the differences between the properties, which variables contribute the most to the difference and which variables are correlated with each other or completely independent from each other. According to the length of the loading vectors and the angle between the variable vectors, the significance of the individual variables and the correlation between the variables, respectively, could be determined [41]. 

In this study, using the covariance matrix with autoscaling, PCA of the total intensities of all characterized phenolic compounds was applied with the aim of separating the most suitable unions and determining the relationship between variables in the initial matrix of 27 homeo- and heteroplastic unions and 19 phenolic compounds, TPC and RSA.

**Figure 3:**
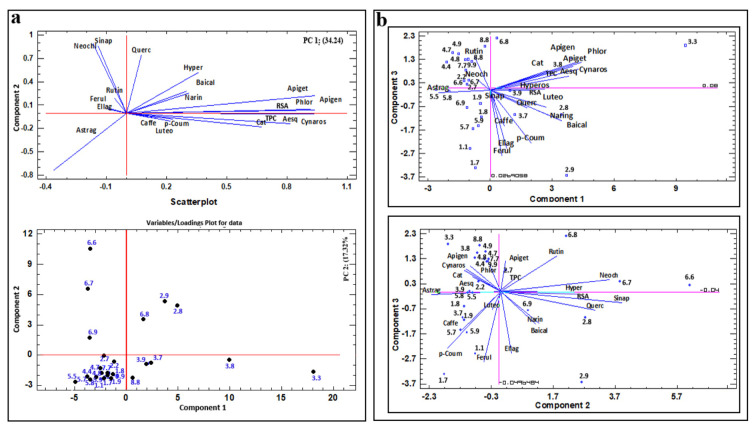
(**a**) The principal component analysis (PCA) projection of variables (polyphenols compounds) into a plane consisting of PCA components PC1 and PC2. The PCA component 1 represents 34.24% of the total data variability. The component PC2 represents 17.32% of the total data variability; (**b**) PCA projection of PC1/PC3 samples/scores plot of callus union and PC2/PC3 biplot of callus union samples. The PC3 component represent 12.09% of total data variability.

The six PCs explain 88.46% of the variation of the data set. The first principal component (PC1) accounted for 34.24% of the total variance, the second for 17.32%, the third (PC3) component for 12.09%, the fourth (PC4) for 11.09%, the fifth (PC5) for 8.47% and the sixth (PC6) for 5.26%. PCA’s samples/scores plot (Figure 3) shows the dependencies of the first three main components—PC1, PC2 and PC3, as well as significantly correlated variables in the same direction (revealing five groups). The first encompasses TPC, RSA, apigenin, apigetrin, aesculin, phlorizine, catechin and cynaroside. In the second group are caffeic acid, *p*-coumaric and luteolin, in the third are baicalein, hyperoside and naringenin. The fourth group consists of rutin, ferulic acid and ellagic acid, while in the fifth are neochlorogenic acid and sinapic acid. Due to the lower eigenvalue of the PC3, loadings and scores on PC1/PC3 and PC2/PC3 plots are different and spatial distribution of vectors has changed. It is just evident that the angle between vectors from the previously formed group (TPC, RSA, apigenin, apigetrin, aesculin, phlorizine, catechin, and cynaroside) has even decreased in the PC1/PC3 plot, which explains very tight correlation between those loadings. Caffeic acid and *p*-coumaric acid, and baicalein and naringenin form groups in all three plots, suggesting significant correlation.

The biggest group (those showing compatibility) comprised 1.1, 1.8, 1.9, 1.7, 4.4, 4.7, 4.8, 4.9, 5.5, 5.8, 5.7 and 5.9 unions were located in the third quadrant of the PC1/PC2 plot, while unions that are showing incompatibility are scattered in the remaining three quadrants (Figure 3).

## 3. Discussion

In grafting research, cell staining at the contact surface is considered an important factor indicating potential incompatibility. Histochemical analysis showed staining in the section of graft unions and confirmed the accumulation of phenols at the cellular level of the unions. According to Pina et al. [26], histochemical staining for phenolic compounds showed that the undifferentiated callus cells at the graft interface of incompatible unions accumulated phenols. Further, the authors concluded that contents and affinity of the safranine/methylene blue staining of vacuoles or cell walls may suggest different storage sites for phenolic compounds at early stages of graft development. The results obtained by fusing callus tissues of *P. mahaleb*, as rootstock, and *Sato-zakura* cherries, show that accumulation of phenol deposits at the union and the presence of phenolic compounds in callus cells were higher than in cells from potently compatible combinations (*P. serrulata* as rootstock). The accumulation of particular phenols immediately above the union in incompatible apricot/plum graft unions also indicated a typical symptom of a local type of incompatibility [37]. A similar conclusion is reached by Nito et al. [11] who analyzed compatible and incompatible unions of species in the genus *Citrus*. In Ermel et al. [9] who studied incompatibility between quince and pear, and Errea et al. [24] who studied apricot and different *Prunus* rootstocks incompatibility, the junction between rootstock and scion tissue was stained with toluidine blue of different intensities as a result of modified polyphenolic content in the scion tissue near the union. The callus of cultivars ‘Amanogawa’ and ‘Kiku-shidare-zakura’ with the callus of *Prunus serotina* (one of the two species selected for a negative control of the compound) did not completely give the expected response of incompatible fusion. The development of successful callus unions with *P. serotina*, which was considered as a negative control, has been shown to be a potentially compatible rootstock.

The unions of the same cultivars with the *Prunus avium* (most frequently used as a rootstock for grafting Japanese ornamental cherry trees) confirmed the existence of certain anomalies with the unions. These symptoms can be associated with weak union forms or some kind of partially or delayed incompatibility. According to the analyses concerning these two cultivars, better results were achieved with the rootstock *Prunus serrulata*, and this Japanese cherry taxon could be used as interstock, as an alternative. The oxidation processes and difference in the stain of phenols begin when phenols are found free in the cell cytoplasm or when accumulate along the cell wall [24,37]. All these processes are related to the toxicity of phenolic compounds and their possibility of altering surrounding cells and tissues [20]. Data obtained through histological analysis confirmed that the presence of phenols in incompatible and compatible callus unions, as well as their impact on the success of callus unions, can be considered as early indicators of graft incompatibility. Long lasting (6–7 years), costly and labor and resource intensive field experiments could be avoided by applying histochemical and *in vitro* techniques. A response concerning the callus incompatibility could be expected for 6–8 months.

Numerous authors quantified polyphenols and analyzed the difference in amounts that occur in callus unions of two grafting components, due to the possibility that it may be the cause of metabolic dysfunction at the union [13,29,35,37,42,43]. Peer et al. [44] indicated that flavonoids are most likely functioning as endogenous regulators of polar auxin transport because some of flavonoids compete for binding sites on membranes. Quercetin, and apigenin can outperform auxin in binding to plasma membrane sites. In the abovementioned studies, all these processes were recorded directly in the tissue of the rootstock and scion, while in *in vitro* unions the quantity of quantified polyphenols is much lower. Among the compounds from the group of flavonols, for which a negative role in the formation of the union was confirmed, in our callus union studies with Japanese cherries, an increase in concentration was recorded for quercetin in the union with *P. mahaleb* (3.7, 3.8, 3.9), ‘Pyrodwarf’ (6.7, 6.8, 6.9), ‘Colt’/‘Kanzan’ and ‘Colt’/‘Kiku-shidare-zakura’ (2.8, 2.9). 

According to the studies by Cooman et al. [29], Musacchi et al. [36] and Usenik et al. [13], the accumulation of catechins above the graft union can be taken as a biochemical marker of graft incompatibility. The findings of the current study confirmed this in heteroplastic combinations 3.7, 3.8, 3.9, 1.7 and 1.8 and homeoplastic unions 3.3, 8.8, 7.7 and 1.1. It should be taken into consideration that the concentration of catechin increased very rapidly as a result of tissue injury after grafting and even small quantities of phenols could be enough to produce abnormality in the intersection [30,31]. The phenols released from the vacuole pass into the cytoplasmic matrix where the oxidation process causes growth dysfunction or induces hormonal imbalance [20,45]. 

Based to the obtained measurements, a completely identical pattern of increase and decrease of baicalein and naringenin in the unions of Japanese cherries and domestic rootstocks was recorded. The increase occurs in all three cultivars in unions with cultivars ‘Colt’ and *P. mahaleb*. The role of naringenin as a growth inhibitor that stimulates IAA oxidase by promoting gibberellin activity is well known [46]. In xylem tissue, prunin and naringenin, which accumulate at the heteroplastic union, can affect the closure of the injury site.

Phenolic acids whose presence has been detected in callus unions are: *p*-coumaric acid, caffeic, ferulic, ellagic, sinapic and neochlorogenic acid which are also detected in calluses of black chokeberry [47], strawberry [48], raspberry [49] and grapevine [50]. The most common of all hydroxycynnamic phenolic acids occurring in fusions in varying concentrations is *p*-coumaric acid. Increasing content of this compound is recorded in the unions 1.7, 2.9, 5.9 and 6.9. Value of *p*-coumaric acid in homeoplastic combinations was significantly lower than in heteroplastic unions. As far as in the unions of all three cultivars of Japanese cherries with the cultivar ‘Colt’ and ‘Pyrodwarf’ combinations, a significant increase in sinapic acid was obtained. The concentration of caffeic acid was not identified in Japanese cultivars and ‘Colt’ combinations, but a significantly higher concentration of this acid was marked in union 1.7. A significant increase in ferulic acid was shown in unions 5.7, 5.9 and 2.9.

In the studies of *in vitro* culture by micrografting with *Eucalyptus gunnii*, Cooman et al. [29] obtained statistically significant differences in the *p*-coumaric acid content in incompatible unions. Twenty days after grafting, in addition to catechins, the content of *p*-coumaric acid increased. In contrast to the increase in the concentration of this phenolic compound in incompatible unions, the same study stated their decrease in compatible unions. Studies have shown that in species incompatible with the *Prunus* genus [13,30], an increase in *p*-coumaric acid was recorded. In addition, it was also pointed out that special attention should be paid to the concentrations of other identified and unidentified phenolic compounds, whose behavior pattern in the junction is similar to the one of catechin and *p*-coumaric acid. After analysis of the polyphenolic compounds the presence of sinapic and neochlorogenic acid in incompatible unions 6.7, 6.8 and 6.9 can be identified as markers, primarily present only in pear callus tissue. Furthermore, apigenin was present only in *P. mahaleb* unions, and ellagic, caffeic, ferulic and sinapic acids were found in higher concentrations only in calluses of ‘Colt’ and *P. mahaleb* rootstocks but not in scion. We also took into consideration that ellagic acid was only isolated in heteroplastic combinations with *P. avium* (1.1, 1.7, 1.8), without recognition of this compound in homeoplastic *Sato-zakura* callus unions. According to Canas et al. [31], sinapic acid is present in lower amounts in incompatible than in compatible unions in the genus *Vitis* spp., although according to Musacchi et al. [36], the high concentration of ferulic and sinapic acids in the callus union is most likely related to lignin biosynthesis. Canas et al. [31] also confirmed that phenolic acids that are potentially seen as indicators of incompatibility are catechin, epicatechin, ferulic and caffeic acid. Nevertheless, it should be borne in mind that caffeic acid is an inhibitor of the IAA oxidase enzymes and therefore increases the IAA levels in tissues [51]. 

Union samples analyzed in this experiment were well distinguished with a PCA. Based on this it was clarified that all examined unions showed different chemical profiles. Previously, PCA was applied for classification in forestry and fruit science based on their genotype, varieties, bioactivity, volatiles and antioxidant compounds [52,53,54]. 

Within the PC1/PC2 map and space, the highest density of unions is located in the third quadrant. The unions in this part of the PC plot are those with *P. serrulata* (4.4, 4.7, 4.8, 4.9), *P. serotina* (5.5, 5.8, 5.9, 5.7) and *P. avium* (1.1, 1.8, 1.9, 1.7), which have low concentrations of total phenol content, and decreased antioxidant activity, catechin, apigetrin, cynaroside, phlorizine, aesculin and apigenin (Figure 3). These unions were affected by decreased concentrations of rutin and hyperoside compared to its homeoplastic unions. The unions that show incompatibility (2.8, 2.9, 3.7, 3.8, 3.9, 6.7, 6.8, 6.9) are scattered in the remaining three quadrants. Apigenin, phlorizin, apigetrin, cynaroside, aesculin, catechin, TPC and RSA have the highest impact on the separation of unions along the PC1 axis. The union group that was the most influenced by caffeic acid, *p*-coumaric acid, luteolin, catechin, apigenin and aesculin includes all unions with *P. mahaleb* (3.8, 3.7, 3.9, 3.3). According to the studies of Cooman et al. [29] content of *p*-coumaric acid and catechin increased in incompatible unions and accumulation of catechins above the graft union can be taken as a biochemical marker of graft incompatibility combinations [13,36]. Higher contents of quercetin, rutin, ferulic acid, neochlorogenic acid and sinapic acid were the most influential in distinguishing unions 6.6, 6.7, 6.8 and 6.9 from the other. The specific polyphenolic profile with influences of ellagic acid, naringenin, baicalein and hyperoside contribute the most to the formation of unions 2.7, 2.8 and 2.9. 

The unions recognized as exposed to the impact of the above phenolic compounds with a negative sign after grouping are unions with *P. serrulata* (4.4, 4.7, 4.8, 4.9), *P. serotina* (5.5, 5.8, 5.9, 5.7) and *P. avium* (1.1, 1.8, 1.9, 1.7), and the variables with the most significant contribution identified by the vector of latent variables indicate low concentrations of total phenol content, and decreased antioxidant activity, catechins, apigetrin, cynaroside, phlorizine, aesculin and apigenin (Figure 3). The only polyphenol component that was present in compatible unions, but absent from incompatible ones, was flavonol astragalin. Neochlorogenic acid and sinapic acid first, and partly quercetin, were the most significant in differentiating unions 6.9, 6.8 and 6.7 from the other unions, thus those three phenolic compounds could be markers of incompatibility for *Pyrus* ‘Pyrodwarf’ rootstock. The specific polyphenolic profile with high level of ellagic acid, baicalein and naringenin could be used to point out unstable joints with ‘Colt’. Finally, caffeic, apigenin and luteolin were the compounds that indicated incompatible unions with *P. mahaleb.* Besides, *p*-coumaric and ferulic acid could be biomarkers for possible delayed incompatibility between *P. avium* as rootstock for *Sato-zakura* cherries (unions 1.7 and 1.9).

In addition to the results of the ratios of polyphenolic compounds in different homeo- and heteroplastic compounds, Loupit and Cookson [55] underlined the possibility of selection of appropriate rootstocks for grafting by isolating specific products of secondary metabolism that would be identified as marker of successfully grafting fusion.

## 4. Materials and Methods

### 4.1. Chemicals

The reagents acetonitrile and formic acid (both of MS grade), gallic acid, Folin–Ciocalteu reagent and methanol (HPLC grade) were purchased from Merck (Darmstadt, Germany). Ultrapure water was used to prepare standard solutions and blanks (ThermoFisher TKA MicroPure, 0.055 µS/cm, Bremen, Germany). Syringe filters (13 mm, PTFE membrane 0.45 μm) were purchased from Supelco (Bellefonte, PA, USA) and phenolic standards and 2,2-diphenyl-1-picrylhydrazyl radical were purchased from Fluka AG (Buch, Switzerland).

### 4.2. In Vitro Callus Cultures

**Figure 4:**
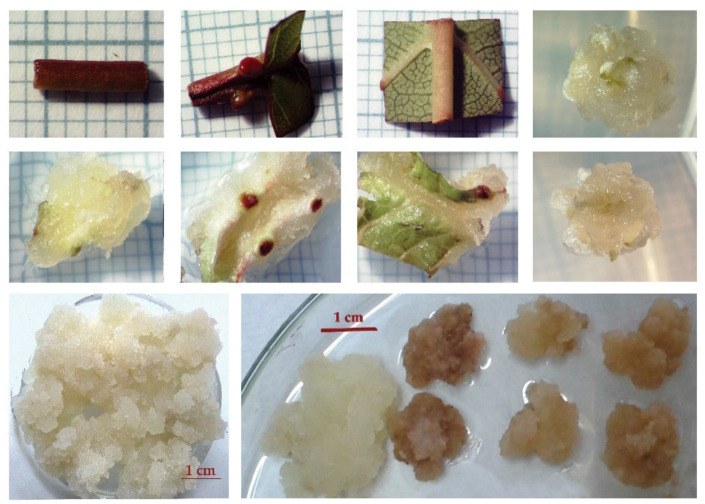
*In vitro* explants and callus induction (in the first and second row): part of petiole, leaf base, part of lamina and axillary bud. Friable callus preparing for analysis (third row).

Plant tissues were collected from four-year-old nursery stock of *P. serrulata* ‘Amanogawa’, ‘Kanzan’ and ‘Kiku-shidare-zakura’ from ‘Manojlović nursery’ in Počekovina, Trstenik (43°34′53.93″ N, 21°6′4.20″ E), and three-year-old rootstock of *P. avium*, *Prunus* ‘Colt’ and *Prunus mahaleb* grown at the open field in nursery ‘Mind’ (Ljubić near the Čačak, 43°53′45.93″ N, 20°22’55.14″ E). *Prunus serrulata* and *Prunus serotina* were collected from 5-year-old trees from Arboretum of the Faculty of Forestry at the University of Belgrade (44°46′58.53″ N, 20°25′23.97″ E). According to Skočajić et al. [19], elongated branches, developed in the spring within the period of 4–5 weeks after the beginning of flowering, were used for callus induction. A petiole, leaf base, part of lamina and part of a stem with an axillary bud were used as explant sources for callogenesis (Figure 4).

The 0.65% agar medium was adjusted to pH 5.8 and then sterilized by autoclaving at 121 °C and 1.5 atm for 20 min. In order to induce a white, fast growing friable callus tissue, different mediums M&S [56] and SH [57] supplemented with different concentrations of 2.4-D, NAA, IBA and BAP [19]. The concentration (mgL^−1^) and composition of auxins 2.4-D, NAA, IBA and cytokinin BAP in different media are summarized in Table 3.

All the explants were placed in glass jars (vessel) Ø 5.5 × 5.5 cm with 25 mL of the medium and maintained at 23 ± 2 °C in the dark. The fragments of induced friable callus tissues were cut into small pieces of 4–5 mm wide and subcultured every month on the most appropriate medium under the same conditions of light (dark) and temperature (23 ± 2 °C). 

**Figure 5:**
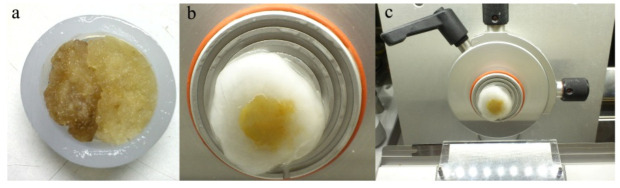
Preparation of callus union for histological analysis: (**a**) the fusion of two calluses in a polyethylene ring; (**b**,**c**) frozen callus union preparing for cutting process on cryotome.

*In vitro* callus unions were established by making clean-cut callus pieces and placing one next to each other in the same conditions as used for callus induction. To prevent callus growth on the opposite side of the fusion, callus combinations for histochemical analysis were inserted into a 9 mm diameter polyethylene ring (Figure 5a). 

### 4.3. Preparation of Temporary Samples

As phenolic compounds have the ability to autofluorescence, their identification and localization were performed on an epifluorescence microscope (LEICA DMSL microscope with UV filter A, excitation filter BP 340–380 nm/suppression filter LP 425 (Leica Microsystems Wetzlar GmbH, Vienna, Austria). Temporary samples for fluorescence microscopy were cut with a cryotome (Kryostat microtome/drehbares/halbautomatisch/ Stehend Slee MEV) after freezing. In this procedure, 30 µm thick sections were obtained (Figure 5b,c). The sections were placed on a plate and covered with a glass cover with the addition of a drop of water. The aqueous solution of callus section was examined using fluorescence microscopy (Leica manufactured DMLS upright microscope; excitation filter BP wavelength 450–490 nm). 

### 4.4. Paraffin-Embedded Samples

Rings with calluses in molds were fixed in fixative FAA (10 parts 37–40% formaldehyde, 70 parts 95% ethanol, 15 parts water and 5 parts acetic acid) for 24 h and then rinsed with 70% alcohol. Dehydration was performed in an automatic tissue processor LEICA TP1020 by passing the material through increasing concentrations of ethanol all the way to absolute ethanol (50, 70 and 96%). After infiltration in ethanol (1:1) overnight, the specimens were embedded in Historesin 500-LK and sectioned into 10 µm sections [24] in Ultracut E ultramicrotome (Reichert-Jung, Heidelberg, Germany). Preparations were then stained with toluidine blue, safranine and methylene blue for detecting phenolic deposits. The histological observations were carried out 21 days after callus fusion from both homografts and heterografts combinations. 

### 4.5. Callus Preparation for Chemical Analyses of Polyphenol Compounds

**Figure 6:**
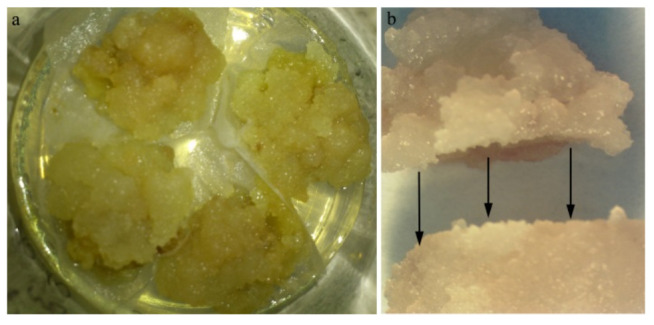
(**a**) Pieces of callus in agar medium; (**b**) flatting part of two callus a prior the union.

Callus fused as a homeo- or heteroplastic combination was placed on a nutrient medium according to a partially modified procedure [24]. In order to prevent possible agar adhesion to the callus sample, the permeable filter paper was placed between the agar and callus (Figure 6a). Fusion was established between callus pieces (5 mm wide) by making clean-cuts on both pieces of callus and placing one on top of the other (Figure 6b). Callus unions of all homeo- or heteroplastic tissue combinations were placed in glass jars (vessel) Ø 5.5 × 5.5 cm with 25 mL of the medium. The number of replicates was 3 × 4 samples of each scion/stock combination. The different combinations were maintained in a growth chamber at 22 ± 2 °C with a 16 h photoperiod of 17 mol m^−2^ s^−1^ provided by cool white fluorescent tubes. 

The combinations of callus union are marked with two numbers, the first one designating the type of rootstock and the second one the type of scion. The markings used to designate the callus of the species of scions and rootstocks in determining the success of callus fusions are given Table 4. 

### 4.6. Determination of TPC and DPPH Radical Scavenging Activity

After 21 days of growth on a medium, 5 g of each sample of callus were used to prepare extracts according to the method described by Pavlović et al. [58]. A mixture of methanol/water (70/30, *v*/*v*) containing 0.1% HCl was used as extraction solvent. These solutions were used for further TPC and RSA analyses.

The total phenolic content was obtained using the Folin–Ciocalteu reagent. The TPC values were expressed as grams of gallic acid equivalent (GAE) per kilogram fresh weight (FW). The radical scavenging activity of the callus extracts was measured employing the DPPH˙ method of Pavlović et al. [58]. The results are expressed as millimoles of Trolox equivalents per kilogram of fresh sample (mMTE/kg).

### 4.7. LC−MS Analysis of Polyphenolic Compounds

A mixture of each phenolic standard was prepared in methanol as 1000 mg/L stock solution. Dilution of the stock solution with methanol yielded working solutions at concentrations of 0.025, 0.050, 0.100, 0.250, 0.500, 0.750 and 1.000 mg/L. They were kept in the dark at 4 °C. Calibration curves were obtained by plotting the peak areas of the standards against their concentration.

The polyphenolic compounds were separated using a Dionex Ultimate 3000 UHPLC system connected to a TSQ Quantum Access Max triple quadruple (QqQ) mass spectrometer (Thermo Fisher Scientific, Bremen, Germany). Separation was achieved at 40° C on a 100 × 2.1 mm i.d., 1.7 μm, Syncronis C18 analytical column (Thermo Fisher Scientific, Bremen, Germany). The mobile phase consisted of (A) water + 0.1% formic acid and (B) acetonitrile. The gradient program was as follows: 0.0–10.0 min, 5–95% B; 10.0–12.0 min, 95% B; 12.0–12.2 min, 95–5% B; and 12.2–15.0 min, 5% B. The injection volume for all samples was 5 µL, and the flow rate was 300 µL/min.

Parameters of the ion source were as follows: source voltage 5 kV, capillary voltage −40 V, tube lens voltage −80 V, capillary temperature 275 °C, sheath and auxiliary gas flow (N_2_) 42 and 11 (arbitrary units). The mass spectrometer was operated in negative mode from *m/z* 100 to 1000. In order to quantify the polyphenols for each standard; a molecular ion and the two most intense fragments from the MS^2^ spectrum were recorded in particular, and collision energies were specified in Table S1. Detailed chromatographic and MS parameters were previously published by Gašić et al. [59].

Xcalibur software (version 2.2, Thermo Fisher Scientific, Bremen, Germany) was used for instrument control. Phenolics were quantitated according to the corresponding spectrometric characteristics of reference standards and total contents of all compounds were obtained by plotting the peak areas and expressed as mg/L.

### 4.8. Statistics Analysis

Data for all measurements made in triplicate are expressed as the mean ± standard error (SE). One-way analysis of variance (ANOVA) was used to evaluate the experimental data, followed by Tukey’s test to detect significant differences (*p* ≤ 0.05) between the mean values. These analyses were performed with the statistical program MS Excel (Microsoft Office 2007 Professional). PCA was carried out employing the program Statgraphics Plus 4.0 (Manugistics, Inc., Rockville, MD, USA). All data were group-scaled prior to PCA. 

## 5. Conclusions

Based on significant differences between homeoplastic and heteroplastic combination of callus unions we can conclude that within rootstocks tested in our research, the most compatible with *Sato-zakura* cherries is cultivar *P. serrulata*. With *P. avium*, especially in heteroplastic combinations 1.7 and 1.9, we can expect problems with delay incompatibility. Rootstocks *P. mahaleb* and Pyrus ‘Pyrodwarf’ are unsuitable for *Sato-zakura* cherries, while ‘Colt’ is not good for cultivars ‘Kanzan’ and ‘Kiku-shidare-zakura’. Among all quantified polyphenols, all incompatible unions showed elevated levels of quercetin, which can use as a universal marker for improper grafting. Markers for unstable joints with ‘Colt’ and *P. mahaleb* could be underlined baicalein and naringenin and for *Pyrus* ‘Pyrodwarf’ sinapic and neochlorogenic. After this experiment, a field study should be organized in order to follow accumulation of polyphenols in both parts of *Sato-zakura* cherry unions for longer periods, and make a correlation between field and lab results. 

Summarizing the results of the performed research, it can be ascertained that the applied method of *in vitro* callus fusion may be considered an efficient way of receiving a relatively quick response concerning the potential compatibility of two grafting components and the applicability of the method on other species in forestry and agriculture. By applying this technique, field experiments where a response concerning the early (or even delay) grafting incompatibility could be expected for several years, could be avoided. The field testing of the best graft combinations is time, space and the money consuming process, thus this kind of experiment could speed up introduction of new cultivars and rootstocks and their combinations. Besides showing good grafting combination, this method can be used to select ‘undesirable’ calluses which, due to high polyphenols synthesis, can be used as additives for functional food production.

## Figures and Tables

**Table 1:** Average of total phenolic content (TPC) and the radical scavenging activity (RSA) ± standard error in callus unions (analyzed cultivars grafted on different rootstocks).

Callus Union		TPC (gGAE/kg)		RSA (mmol TE/kg)	
Marks	species/cultivar	F = 708.74	*p* = 0.00	F = 239.26	*p* = 0.00
1.1.	avium/avium	0.28 ± 0.010 ^q^*		4.715 ± 0.045 ^k^	
1.7.	avium/‘Amanogawa’	0.425 ± 0.005 ^p^		5.96 ± 0.040 ^ih^	
1.8.	avium/‘Kanzan’	0.65 ± 0.000 ^lk^		6.415 ± 0.015 ^hg^	
1.9.	avium/‘Kiku-shidare-zakura’	0.535 ± 0.005 ^onm^		6.38 ± 0.000 ^hg^	
2.2.	‘Colt’/‘Colt’	0.51 ± 0.035 ^on^		8.375 ± 0.235 ^f^	
2.7.	‘Colt’/‘Amanogawa’	1.205 ± 0.005 ^ji^		5.965 ± 0.015 ^ih^	
2.8.	‘Colt’/‘Kanzan’	1.445 ± 0.005 ^gf^		9.58 ± 0.000 ^e^	
2.9.	‘Colt’/‘Kiku-shidare-zakura’	1.165 ± 0.005 ^j^		9.925 ± 0.095 ^e^	
3.3.	mahaleb/mahaleb	3.77 ± 0.020 ^b^		11.795 ± 0.135 ^d^	
3.7.	mahaleb/‘Amanogawa’	4.24 ± 0.120 ^a^		12.875 ± 0.015 ^cb^	
3.8.	mahaleb/‘Kanzan’	2.275 ± 0.045 ^d^		13.585 ± 0.115 ^b^	
3.9.	mahaleb/‘Kiku-shidare-zakura’	2.425 ± 0.005 ^c^		12.46 ± 0.060 ^dc^	
4.4.	serrulata/serrulata	0.425 ± 0.015 ^po^		3.325 ± 0.025 ^l^	
4.7.	serrulata/‘Amanogawa’	0.665 ± 0.005 ^lk^		3.43 ± 0.010 ^l^	
4.8.	serrulata/‘Kanzan’	0.58 ± 0.000 ^nmlk^		3.355 ± 0.025 ^l^	
4.9.	serrulata/‘Kiku-shidare-zakura’	0.685 ± 0.005 ^k^		3.445 ± 0.005 ^l^	
5.5.	serotina/serotina	0.187 ± 0.000 ^r^		5.78 ± 0.000 ^jih^	
5.7.	serotina/‘Amanogawa’	0.475 ± 0.005 ^on^		6.245 ± 0.015 ^hg^	
5.8.	serotina/‘Kanzan’	0.355 ± 0.005 ^p^		6.455 ± 0.115 ^hg^	
5.9.	serotina/‘Kiku-shidare-zakura’	0.355 ± 0.005 ^p^		6.845 ± 0.085 ^g^	
6.6.	Pyrus ‘Pyrodwarf’/Pyrus ‘Pyrodwarf’	1.29 ± 0.000 ^ih^		5.13 ± 0.060 ^kj^	
6.7.	Pyrus ‘Pyrodwarf’/‘Amanogawa’	2.14 ± 0.040 ^e^		15.64 ± 1.340 ^a^	
6.8.	Pyrus ‘Pyrodwarf’/‘Kanzan’	1.46 ± 0.040 ^f^		16.25 ± 0.230 ^a^	
6.9.	Pyrus ‘Pyrodwarf’/‘Kiku-shidare-zakura’	1.34 ± 0.020 ^hg^		15.685 ± 0.115 ^a^	
7.7.	‘Amanogawa’/‘Amanogawa’	0.56 ± 0.000 ^nml^		3.595 ± 0.005 ^l^	
8.8.	‘Kanzan’/‘Kanzan’	0.625 ± 0.005 ^mlk^		5.23 ± 0.020 ^kji^	
9.9.	‘Kiku-shidare-zakura’‘Kiku-shidare-zakura’	0.555 ± 0.005 ^nml^		3.3 ± 0.010 ^l^	

* Different letters in the same row denote a significant difference according to Tukey’s test, *p* < 0.05.

**Table 2:** The content of polyphenol compounds (mg/L DW) in analyzed callus unions of cultivars and different rootstocks. * Different letters in the line indicate statistically significant differences in the content of a specific phenolic compound by Tukey’s test at *p* ≤ 0.05; nf—not found.

	1a ^†^	1b					1c					1d	2	3					
Callus Union	Cat ^‡^	Aesc	Querc	Rutin	Hyper	Astrag	Cynaros	Apiget	Luteo	Apigen	Baical	Narin	Phlor	*p*-Coum	Caffe	Ferul	Ellag	Sinap	Neoch
1.1.	1.009 ^a^*	0.368 ^g^	0.051 ^d^	0.285 ^a^	0.164 ^hi^	0.032	0.258 ^a^	0.012 ^a^	nf	nf	0.025 ^a^	0.048 ^a^	nf	0.146 ^h^	0.390 ^d^	0.101 ^g^	0.018 ^c^	nf	nf
1.7.	1.445 ^e^	0.273 ^d^	0.071 ^fg^	0.283 ^a^	0.088 ^b^	0.032	0.583 ^d^	0.012 ^a^	nf	nf	0.040 ^c^	0.062 ^c^	0.030 ^a^	0.216 ^l^	0.398 ^e^	0.127 ^i^	0.015 ^b^	nf	nf
1.8.	2.345 ^i^	0.311 ^e^	0.058 ^e^	0.291 ^a^	0.102 ^d^	0.032	0.733 ^f^	0.012 ^a^	nf	nf	0.054 ^d^	0.079 ^d^	0.051 ^d^	0.127 ^f^	0.376 ^c^	0.067 ^dd^	0.009 ^a^	nf	nf
1.9.	1.233 ^c^	0.304 ^e^	0.046 ^cd^	0.299 ^ab^	0.105 ^d^	0.032	1.453 ^k^	0.012 ^a^	0.175 ^c^	nf	0.045 ^c^	0.067 ^c^	0.052 ^d^	0.147 ^h^	0.367 ^a^	0.059 ^c^	nf	nf	nf
2.2.	2.114 ^g^	0.399 ^h^	nf	0.279 ^a^	0.076 ^a^	nf	0.518 ^d^	0.012 ^a^	nf	nf	0.033 ^b^	0.052 ^b^	0.030 ^a^	0.064 ^a^	nf	nf	0.015 ^b^	0.114 ^a^	nf
2.7.	1.22 ^c^	0.227 ^b^	0.035 ^ab^	0.282 ^a^	0.081 ^b^	nf	0.690 ^e^	0.013 ^a^	nf	nf	0.047 ^c^	0.070 ^c^	0.036 ^b^	0.080 ^c^	nf	nf	nf	0.255 ^b^	nf
2.8.	1.29 ^c^	0.379 ^g^	0.162 ^j^	0.288 ^a^	0.168 ^i^	nf	1.659 ^l^	0.016 ^a^	0.212 ^g^	nf	0.147 ^g^	0.185 ^g^	0.118 ^h^	0.066 ^a^	nf	nf	0.015 ^b^	0.266 ^b^	nf
2.9.	1.365 ^d^	0.438 ^i^	0.196 ^k^	0.288 ^a^	0.128 ^f^	nf	1.398 ^j^	0.014 ^a^	0.338 ^i^	nf	0.156 ^h^	0.197 ^h^	0.080 ^g^	0.149 ^h^	nf	0.078 ^e^	0.019 ^c^	0.513 ^c^	nf
3.3.	2.914 ^j^	0.923 ^l^	0.115 ^h^	0.289 ^a^	0.160 ^h^	nf	9.743 ^p^	0.025 ^b^	0.211 ^g^	0.179 ^b^	0.090 f	0.122 ^f^	0.261 ^j^	0.194 ^k^	0.370 ^ab^	0.060 ^c^	nf	nf	nf
3.7.	1.162 ^b^	0.243 ^bc^	0.061 ^e^	0.283 ^a^	0.095 ^c^	nf	3.391 ^n^	0.013 ^a^	0.187 ^d^	0.023 ^a^	0.058 ^de^	0.086 ^e^	0.085 ^g^	0.191 ^k^	0.364 ^a^	0.052 ^b^	nf	nf	nf
3.8.	2.195 ^gh^	0.457 ^j^	0.073 ^g^	0.290 ^a^	0.130 ^f^	nf	4.881 ^o^	0.019 ^ab^	0.173 ^c^	0.089 ^b^	0.063 ^e^	0.091 ^e^	0.154 ^i^	0.097 ^e^	0.364 ^a^	0.043 ^a^	nf	nf	nf
3.9.	2.878 ^j^	0.22 ^b^	0.057 ^e^	0.290 ^a^	0.092 ^c^	nf	2.446 ^m^	0.013 ^a^	0.193 ^e^	0.023 ^a^	0.057 ^d^	0.082 ^de^	0.061 ^e^	0.171 ^j^	nf	0.048 ^b^	nf	nf	nf
4.4.	nf	0.255 ^c^	0.036 ^ab^	0.292 ^a^	0.098 ^c^	0.032	0.618 ^e^	0.012 ^a^	nf	nf	0.035 ^b^	0.057 ^b^	0.047 ^c^	0.066 ^a^	nf	nf	nf	nf	nf
4.7.	1.21^c^	0.250 ^c^	0.037 ^ab^	0.299 ^ab^	0.097 ^c^	0.032	0.664 ^e^	0.012 ^a^	nf	nf	0.034 ^b^	0.055 ^b^	0.055 ^d^	0.066 ^a^	nf	nf	nf	nf	nf
4.8.	1.25 ^c^	0.268 ^d^	0.035 ^ab^	0.290 ^a^	0.099 ^cd^	0.032	0.902 ^h^	0.013 ^a^	nf	nf	0.032 ^b^	0.054 ^b^	0.060 ^e^	0.066 ^a^	nf	nf	nf	nf	nf
4.9.	1.21 ^c^	0.262 ^cd^	0.048 ^cd^	0.295 ^a^	0.112 ^e^	0.032	0.833 ^g^	0.013 ^a^	0.154 ^a^	nf	0.039 ^bc^	0.063 ^c^	0.055 ^d^	0.066 ^a^	nf	nf	nf	nf	nf
5.5.	nf	0.220 ^b^	nf	0.296 ^a^	0.100 ^cd^	0.031	0.229 ^a^	0.012 ^a^	nf	nf	0.024 ^a^	0.044 ^a^	nf	0.086 ^cd^	0.369 ^ab^	0.047 ^b^	nf	nf	nf
5.7.	nf	0.310 ^e^	0.043 ^c^	0.294 ^a^	0.113 ^e^	0.031	0.741 ^f^	0.012 ^a^	0.161 ^b^	nf	0.043 ^c^	0.067 ^c^	0.045 ^c^	0.153 ^i^	0.374 ^bc^	0.110 ^h^	nf	nf	nf
5.8.	nf	0.310 ^e^	0.030 ^a^	0.287 ^a^	0.102 ^d^	0.031	0.483 ^c^	0.012 ^a^	nf	nf	0.027 ^a^	0.049 ^ab^	0.037 ^b^	0.082 ^c^	0.366 ^a^	0.044 ^a^	nf	nf	nf
5.9.	nf	0.384 ^gh^	0.039 ^bc^	0.289 ^a^	0.106 ^d^	0.032	0.804 ^g^	0.012 ^a^	0.227 ^h^	nf	0.047 ^c^	0.071 ^c^	0.037 ^b^	0.151 ^i^	0.371 ^b^	0.085 ^f^	nf	nf	nf
6.6.	nf	0.240 ^bc^	0.149 ^i^	0.321 ^b^	0.197 ^j^	nf	nf	0.013 ^a^	nf	nf	0.034 ^b^	0.056 ^b^	0.028 ^a^	0.067 ^a^	0.372 ^b^	0.067 ^d^	nf	0.966 ^d^	0.845 ^d^
6.7.	nf	0.184 ^a^	0.290 ^l^	0.296 ^a^	0.114 ^e^	nf	0.325 ^b^	0.013 ^a^	nf	nf	0.037 ^bc^	0.058 ^bc^	0.040 ^b^	0.063 ^a^	nf	0.041 ^a^	nf	0.333 ^bc^	0.533 ^c^
6.8.	2.22 ^h^	0.345 ^f^	0.057 ^e^	0.305 ^ab^	0.149 ^g^	nf	0.951 ^h^	0.017 ^a^	nf	nf	0.047 ^c^	0.070 ^c^	0.072 ^f^	0.075 ^b^	nf	nf	nf	0.265 ^b^	0.386 ^b^
6.9.	nf	0.189 ^a^	0.065 ^f^	0.280 ^a^	0.082 ^b^	nf	0.421 ^c^	0.012 ^a^	0.193 ^e^	nf	0.044 ^c^	0.069 ^c^	0.029 ^a^	0.133 ^g^	nf	0.048 ^b^	nf	0.292 ^b^	0.298 ^a^
7.7.	1.27 ^c^	0.230 ^b^	0.040 ^bc^	0.296 ^a^	0.098 ^c^	0.032	1.014 ^i^	0.012 ^a^	0.162 ^b^	nf	0.042 ^c^	0.065 ^c^	0.062 ^e^	0.063 ^a^	nf	nf	nf	nf	nf
8.8.	1.80 ^f^	0.491 ^k^	0.044 ^c^	0.299 ^ab^	0.116 ^e^	0.032	1.003 ^i^	0.013 ^a^	0.205 ^f^	nf	0.031 ^b^	0.052 ^b^	0.084 ^g^	0.064 ^a^	nf	nf	nf	nf	nf
9.9.	1.17 ^b^	0.478 ^k^	0.052 ^d^	0.319 ^b^	0.099 ^c^	0.033	1.095 ^i^	0.012 ^a^	0.164 ^b^	nf	0.033 ^b^	0.054 ^b^	0.050 ^cd^	0.091 ^de^	nf	0.049 ^b^	nf	nf	nf

1a ^†^—flavanols, 1b—flavonols, 1c—flavones, 1d—flavanons, 2—dihydrohalcon; 3—phenol acids; ^‡^ Cat—catehin; Aesc—aesculin; Querc—quercetin; Rutin—rutin (quercetin_3-O-rutinoside); Hyper—hyperoside (quercetin_3-O-galactoside); Astrag—astragalin (kaempferol_3-O-glucoside); Cynaros—cynaroside (luteolin_7-O-glucoside); Apiget—apigetrin (apigenin_7-O-glucoside); Luteo—luteolin; Apigen—apigenin; Baical—baicalein; Narin—naringenin; Phlor—phlorizin; *p*-Coum—*p*-coumaric_acid; Caffe—caffeic acid; Ferul—ferulic acid; Ellag—ellagic acid; Sinap—sinapic_acid; Neoch—neochlorogenic acid (5-O-caffeoylquinic acid).

**Table 3:** Composition of callus induction media and concentration of plant growth regulators—PGRs (mgL^−1^) used for callus induction.

PGRs	Culture Medium														
mgL^−1^	MS1	MS2	MS3	MS4	MS5	WP1	WP2	WP3	WP4	WP5	SH1	SH2	SH3	SH4	SH5
BAP	0.5	0.5	0.5	0.5	0.5	0.5	0.5	0.5	0.5	0.5	0.5	0.5	0.5	0.5	0.5
2.4-D	2				2	2				2	2				2
NAA		2		2			2		2			2		2	
IBA			2	0.5	0.5			2	0.5	0.5			2	0.5	0.5

**Table 4:** The number symbol of rootstock and scion *.

Marks	Species/Cultivar
1.	*Prunus avium*
2.	*Prunus* ‘Colt’
3.	*Prunus mahaleb*
4.	*Prunus serrulata*
5.	*Prunus serotina*
6.	*Pyrus communis* ‘Pyrodwarf’
7.	*Prunus serrulata* ‘Amanogawa’
8.	*Prunus serrulata* ‘Kanzan’
9.	*Prunus serrulata* ‘Kiku-shidare-zakura’

* The combination of two numbers (hereinafter) means fusion between the callus of two species/cultivars.

## Data Availability

All data are presented in this manuscript.

## Supplementary Materials

The following are available online at https://www.mdpi.com/article/10.3390/plants10122822/s1, Table S1: The molecular ions and obtained fragments (qualifier and quantifier), with specified collision energies, used at LC/MS quantification method.
