# Supplementary material for: Analysis of Phenolic Compounds for the Determination of Grafts (in) Compatibility Using In Vitro Callus Cultures of Sato-Zakura Cherries
Source: Plants (Basel). 2021 Dec 20;10(12):2822. doi: 10.3390/plants10122822 (PMC8706243; doi:10.3390/plants10122822)
Supplement: Supplementary file 1 [file plants-10-02822-s001.zip › plants-1473723-supplimentary.pdf]

Supplementary Materials: The following are available online at www.mdpi.com/xxx/s1, Table S1: The molecular ions and obtained fragments (qualifier and quantifier), with specified collision energies, used at LC/MS quantification method.

Table S1. The molecular ions and obtained fragments with specified collision energies.

Compounds	Parent Ion, *m*/*z*	Product Ion, *m*/*z* (Collision Energy, eV)
**Catechin**	289.084	203.00 (23); 245.03 (31)
**Aesculin**	339.080	133.09 (44); 177.06 (25)
**Quercetin**	301.026	151.01 (22); 179.00 (20)
**Rutin**	609.197	299.98 (42); 301.20 (32)
**Hyperoside**	463.100	271.00 (43); 300.00 (30)
**Astragalin**	447.008	255.03 (43); 284.03 (29)
**Cynaroside**	447.000	284.04 (40); 285.03 (27)
**Apigetrin**	431.001	239.11 (53); 268.03 (36)
**Luteolin**	285.035	133.05 (30); 150.95 (24)
**Apigenin**	269.032	117.07 (43); 225.09 (23)
**Baicalein**	268.956	233.07 (29); 241.09 (26)
**Naringin**	579.241	151.42 (43); 217.36 (33)
**Phloretin**	273.066	123.26 (26); 167.20 (19)
***p*-Coumaric acid**	163.031	93.12 (39): 119.09 (16)
**Caffeic acid**	179.004	134.00 (13); 135.00 (16)
**Ferulic acid**	193.035	134.06 (19); 178.04 (15)
**Ellagic acid**	300.998	229.00 (27); 284.00 (33)
**Sinapic acid**	223.082	149.21 (36)
**Neochlorogenic acid**	353.103	191.28 (25)
